# Supplementary material for: Microtubule Dynamics Plays a Vital Role in Plant Adaptation and Tolerance to Salt Stress
Source: Int J Mol Sci. 2021 May 31;22(11):5957. doi: 10.3390/ijms22115957 (PMC8199277; doi:10.3390/ijms22115957)
Supplement: Supplementary file 1 [file ijms-22-05957-s001.zip › ijms-1230707-supplementary.pdf]

Supplementary Material

Table 1. Primers used for qRT-PCR.

Gene	Primer Name	Sequence (5′→3′)
AT4G13940 (*SAHH1*)	*AT4G13940*-qRT-F	5′-TGTCACCAAGAGCAAGTTCG-3′
	AT4G13940-qRT-R	5′-TCCAACATCACCATATCCACAG-3′
AT3G52930 (*FBA8*)	*AT3G52930*-qRT-F	5′-AAGACATGGGCAGGTAAAGAG-3′
	AT3G52930-qRT-R	5′-CGAGTGTGGCTTCAGAGTTAG-3′
AT2G47470 (*PDIL2-1*)	*AT2G47470*-qRT-F	5′-AAATCACTCGCTCCCACATAC-3′
	AT2G47470-qRT-R	5′-ACTCCATATTTCTCGCCAAGG-3′
AT1G21750 (*PDIL1-1*)	*AT1G21750*-qRT-F	5′-ACCAAAGCGACTCAAGTGTAG-3′
	AT1G21750-qRT-R	5′-GTCCTGTCTCCTTCGTAAACC-3′
AT5G62700 (*TUB3*)	*AT5G62700*-qRT-F	5′-AAGGAAGCTGAGAACTGTGAC-3′
	AT5G62700-qRT-R	5′-CATGCGATCTGGGTACTCTTC-3′
AT5G44340 (*TUB4*)	*AT5G44340*-qRT-F	5′-GCTGACGAGTGTATGGTTTTG-3′
	AT5G44340-qRT-R	5′-AGGGAAACGAAGACAGCAAG-3′
AT2G29550 (*TUB7*)	*AT2G29550*-qRT-F	5′-GGGCTAAAGGTCACTACACTG-3′
	AT2G29550-qRT-R	5′-AAGGCAGTCACAGTTCTCAG-3′
AT4G20890 (*TUB9*)	*AT4G20890*-qRT-F	5′-TTCCTCGTGCTGTTCTTATGG-3′
	AT4G20890-qRT-R	5′-TTTCGCCCAGTTATTCCCAG-3′
AT5G09810 (*ACT7*)	*AT5G09810*-qRT-F	5′-GAATGGTGAAGGCTGGTTTTG-″
	AT5G09810-qRT-R	5′-AGCATCTTTCTGACCCATACC-3′
AT5G59880 (*ADF3*)	*AT5G59880*-qRT-F	5′-ACAGCAAGAGTGAGAAGCAAG-3′
	AT5G59880-qRT-R	5′-CGACCTGAATTCCGTCTAGTTC-3′
AT4G34050 (*CCoA-OMT1*)	*AT4G34050*-qRT-F	5′-CTGGCTATGGATGTCAACAGAG-3′
	AT4G34050-qRT-R	5′-ATCAAGAACGGGAAGAGCAG-3′
AT4G20260 (*PCAP1*)	*AT4G20260*-qRT-F	5′-CCGGTCACGTTCATATTCGAG-3′
	AT4G20260-qRT-R	5′-CACTTGTTCCTTCGGTTTTGG-3′
AT4G10480 (*NACα4*)	*AT4G10480*-qRT-F	5′-GAGGATGTGAAAGATGGAGACG-3′
	AT4G10480-qRT-R	5′-TCTCGCTTCTACTTTGCTTGG-3′
AT3G62030 (*CYP20-3*)	*AT3G62030*-qRT-F	5′-GAGAAGTTGTGCCTAAAACCG-3′
	AT3G62030-qRT-R	5′-GGAACCCTTGTACCCGTATTTC-3′
AT4G05320 (*UBQ10*)	*AT4G05320*-qRT-F	5′-TGGCATCAACTTTCATTGGA-3′
	AT4G05320-qRT-R	5′-ATGTTGCTCTCCGCTTCTGT-3′
